# Sex differences in morbidity and care-seeking during the neonatal period in rural southern Nepal

**DOI:** 10.1186/s41043-015-0014-0

**Published:** 2015-08-08

**Authors:** Summer Rosenstock, Joanne Katz, Luke C. Mullany, Subarna K. Khatry, Steven C. LeClerq, Gary L. Darmstadt, James M. Tielsch

**Affiliations:** 1 Department of International Health, Bloomberg School of Public Health, Johns Hopkins University, 415 N. Washington St., Baltimore, MD 21231 USA; 2 Nepal Nutrition Intervention Project – Sarlahi, Kathmandu, Nepal; 3 Stanford School of Medicine, Pediatrics - Neonatal and Developmental Medicine, Stanford, CA USA; 4 Department of Global Health, School of Public Health and Health Services, George Washington University, Washington, DC USA

**Keywords:** Gender preference, Neonatal, Morbidity, Care-seeking behavior, Nepal, Early neonatal period, Late neonatal period

## Abstract

**Background:**

South Asian studies, including those from Nepal, have documented increased risk of neonatal mortality among girls, despite their early biologic survival advantage. We examined sex differences in neonatal morbidity and care-seeking behavior to determine whether such differences could help explain previously observed excess late neonatal mortality among girls in Nepal.

**Methods:**

A secondary analysis of data from a trial of chlorhexidine use among neonates in rural Nepal was conducted. The objective was to examine sex differences in neonatal morbidity and care-seeking behavior for ill newborns. Girls were used as the reference group.

**Results:**

Referral for care was higher during the early neonatal period (ENP: 0–7 days old) (50.7 %) than the late neonatal period (LNP: 8–28 days old) (31.3 %), but was comparable by sex. There were some significant differences in reasons for referral by sex. Boys were significantly more often referred for convulsions/stiffness, having yellow body/eyes, severe skin infection, and having at least two of the following: difficulty breathing, difficulty feeding, fever, or vomiting during the ENP. Girls were more often referred for hypothermia. During the LNP, boys were significantly more often referred for having yellow body/eyes, persistent watery stool, and severe skin infection. There were no referral types in the LNP for which girls were more often referred. Less than half of those referred at any point were taken for care (47.0 %) and referred boys were more often taken than girls (Neonatal Period OR: 1.77, 95 % CI: 1.64 - 1.91). Family composition differentially impacted the relationship between care-seeking and sex. The greatest differences were in families with only prior living girls (Pahadi - ENP OR: 1.78, 95 % CI: 1.29 - 2.45 and LNP OR: 1.51, 95 % CI: 1.03 - 2.21; Madeshi - ENP OR: 2.86, 95 % CI: 2.28 – 3.59 and LNP OR: 2.45, 95 % CI: 1.84 – 3.26).

**Conclusions:**

Care-seeking was inadequate for both sexes, but ill boys were consistently more often taken for care than girls, despite comparable referral. Behavioral interventions to improve care-seeking, especially in the early neonatal period, are needed to improve neonatal survival. Addressing gender bias in care-seeking, explicitly and within interventions, is essential to reducing neonatal mortality differentials between boys and girls.

## Background

Boys are at approximately 20 % greater risk of neonatal mortality than girls in high-income countries [1–6]. Most literature attributes this sex difference to underlying biological disadvantages in boys, including less mature lungs at the same gestational age, increased risk of prematurity, respiratory and other infectious morbidities, and higher rates of delivery complications, cesarean section and congenital anomalies [1, 4, 6–10]. Animal models lend further support, showing that testosterone suppresses the immune system, while estradiol and progesterone strengthen innate and humoral immune responses [11–14].

Despite their survival advantage, in South Asia there have been reports of excess neonatal (early, late or both) and infant mortality among girls, with ORs ranging from 1.20 (late neonatal period) in Pakistan to 3.42 (overall neonatal period) in South India [15–19]. Existing literature has pointed to gender preference and differential care-seeking behaviors to explain this inversion of risk. Reports of sex imbalances in countries such as China and India are likely linked to sex selective termination of pregnancy and infanticide [20–22]. Studies have also shown differential perceptions of illness and care-seeking behaviors by care-takers favoring boys [18, 23–26]. Additionally, birth order and prior sibling composition have been shown to be predictors of sex-specific neonatal survival [18, 19].

A secondary analysis using data from the Newborn Washing Study, conducted in Sarlahi, Nepal (described below), examined the effect of biological and environmental factors on the relationship between sex of the newborn and early and late neonatal mortality [19]. The overall neonatal mortality rate within the study population was 32.1/1000 live births. The study found that boys were at greater risk than girls of early neonatal mortality (Relative Risk (RR) (M/F): 1.20 [95 % CI:1.02 – 1.42]), but at lower risk than girls of late neonatal mortality (RR (M/F): 0.70 [95 % CI: 0.52-0.94]) [19]. Consistent with studies conducted in high-income countries, the excess early neonatal mortality among boys was statistically explained by biological factors, primarily respiratory depression, and unconsciousness at or shortly after birth [19]. The excess late neonatal mortality among girls, however, was best explained by environmental factors, including ethnicity and sex-distribution of older siblings, with the greatest differences observed among infants born to families with only prior living girls [19].

The current study aims to further examine sex differences in this population to better understand why girls are at greater risk of mortality during the late neonatal period. Specifically, we examined sex differences in morbidity and care-seeking during the early (birth – 7 days old) and late (8–28 days old) neonatal periods. We also investigated whether differences in care-seeking were associated with specific morbidities and/or prior family composition.

## Methods

### Data collection

The Nepal Newborn Washing Study was carried out between September 2002 and January 2006. It was a pair of nested, prospective, population-based, cluster-randomized, placebo-controlled trials of skin and umbilical cord cleansing with chlorhexidine [27–29]. Briefly, women were recruited in mid-pregnancy, gave oral consent to participate in the study, and were followed until delivery. Technology was not readily available in this geographic area during this study period to determine the sex of the baby during pregnancy. Sex of the infant generally remained unknown until delivery. There were 23 662 live births during the study period. In response to an interim meeting of the Data Safety and Monitoring Board for the trial, systematic collection of referral and care-seeking data were added, and are available for 18 985 of the 23 662 newborns included in the study. Local project workers visited the newborn as soon as possible after birth and again on days 2–6, 8, 10, 12, 14, 21, and 28 to provide trial interventions, track vital status (whether the infant was alive, or had died since the previous visit), and assess the newborn’s health. The assessment, which was repeated at each visit, included a standardized physical exam and maternal report of signs of illness experienced by the neonate since the previous visit. Mothers were asked if the infant had difficulty breathing or feeding since the previous visit, had vomited more than half of what s/he had ingested in the last 24 h, had watery or bloody stools and if so, how often, had experienced convulsions or stiffening of the back, or whether the newborn had been cold to the touch in the previous 24 h. The standardized physical exam included measuring the newborn’s respiratory rate and axillary temperature, recording whether the newborn was experiencing severe chest indrawing, and examining the infant for skin infection. The umbilical cord was examined for signs of infection and the supervisor recorded whether the newborn was conscious and if his/her body or eyes appeared yellow in color. The baby was referred for care if he/she met a standard set of criteria, listing up to three possible reasons (Table 1). The referral variable used in this paper is an indicator of specific morbidities listed above that merited referring the infant for care by the study worker.

**Table 1:** Definitions for Cause-Specific Referral for Care During the Neonatal Period

Reason for Referral	Definition
Unconsciousness	Loss of consciousness observed by field worker or reported by the newborn’s mother
Fever	Temperature of newborn measured at 101 °F or higher by field worker
Pneumonia	Rapid breathing (≧60 breaths/min) AND Fever >101 °F OR Severe chest indrawing observed by the field worker
Convulsions/Stiffness	Convulsions and/or Stiffness of the back observed by field worker, or reported by the newborn’s mother.
Yellow Body/Eyes	Newborn’s body or eyes appeared yellow in color
Persistent Watery Stool	Mother reported that the newborn had loose/watery stools
Blood in Stool	Any blood in the stool reported by the mother or observed by field worker
Hypothermia	Temperature of newborn measured at 96 °F or lower by field worker, OR newborn felt cold to touch
Severe Skin Infection	Field worker observed many pustules or blisters, OR one or more large areas of redness/pus
Cord Infection Level I	Field worker observed moderate or severe redness that extended from the umbilicus to the skin OR moderate or severe swelling that extended from the umbilicus to the skin
Cord Infection Level II	Field worker observed pus coming from the umbilicus AND redness AND swelling of any grade
Any Two in List:	
1) Difficulty Breathing	1) Mother reported or field worker observed that the newborn had difficulty drawing breath in the previous 24 h
2) Vomiting	2) Mother reported that newborn vomited more than half his/her feed
3) Difficulty Feeding	3) Mother reported that the newborn had difficulty feeding or sucking
4) Fever	4) Temperature of newborn measured at 100 °F or higher by the field worker
Other	Referred for other reasons not specified above

Care-seeking data were collected at each home visit by maternal report of care sought for the infant in the previous 24 h. Medical care was sought from a variety of sources including medicine shop/local practitioner, health post (basic clinic not usually staffed by a physician) and hospitals. Home remedy, Dhami Jhankri (traditional healer), Community Health Volunteer/Village Health Worker/Health Assistant, and other treatment were considered para-medical or non-medical care. Supervisors recorded up to three responses for care sought.

### Statistical methods

Statistical analyses were carried out using Stata 11 [30]. Demographic and socio-economic data were compared by sex of the neonate, using cross tabulation and *χ*^2^ tests, to assess comparability between families of infant boys and girls. Analyses were stratified by early and late neonatal periods and ethnicity. The population residing in the study area can be loosely grouped into two overarching ethnicities (Pahadi and Madeshi). The Pahadi ethnic group includes tribal people of Tibeto-Burman descent and those of Aryan descent, who conform to the traditional Hindu caste hierarchy, and migrated from the hills to the low-lying plains. The Madeshi people belong to a culture with roots in the north Indian Gangetic floodplain. As there are important socio-demographic and health indicator differences between these groups (e.g. poorer birth outcomes, higher parity, and lower socio-economic status within the Madeshi group), analyses were stratified by ethnic group.

Binomial variables were created that indicated whether an infant was referred at least once during the neonatal period for any reason, and according to reason for referral. Several care-seeking variables were created including; 1) care sought for any reason, independent of referral status, 2) care-seeking for any reason, given referral, and 3) care-seeking given specific reasons for referral. A continuous care-seeking variable, number of times care was sought for an infant in the neonatal period, was also created. Exploratory analyses to compare care-seeking behaviors by sex of the newborn were conducted using cross tabulation and *χ*^2^ tests for categorical variables and t-tests for continuous variables.

Comparisons between boys and girls in overall care-seeking behaviors, in relation to referral status, were stratified by prior sibling composition and ethnicity. This was done to examine whether the impact of family composition on the relationship between sex of the newborn and care-seeking behaviors was consistent with prior findings showing that family composition differentially impacted the relationship between sex of the newborn and neonatal mortality, with girls born to families with only prior living girls being at greatest risk of death [19]. Families were categorized into three family types for prior sibling composition: 1) Families with no prior living children; 2) Families with prior children, including boys and girls; and 3) Families with prior children, all of whom are girls. Generalized estimating equations (GEE), using a binomial family, logit link with independent correlation, and robust variance were used to calculate odds ratios and 95 % confidence intervals for categorical care-seeking variables, comparing boys to girls. A Gaussian family and identity link with independent correlation and robust variance were used for comparisons of continuous variables by sex of the newborn. GEE was used to adjust for clustered randomization of the parent trial chlorhexidine interventions.

### Ethical review

Women provided verbal consent to participate in the study. The Nepal Health Research Council and the Institutional Review Board at the Johns Hopkins Bloomberg School of Public Health reviewed and approved this study. The trial was registered at www.clinicaltrials.com (trial number: NCT00109616).

## Results

There were no meaningful statistically significant differences in socio-economic or demographic characteristics by sex of the neonate. The only statistically significant difference found was in roof material, and the difference was so small (79.1 % of boys had tile roofs and 77.1 % of girls had tile roofs) that it was deemed unimportant. During the neonatal period, 60.7 % of the newborns in this study population met the criteria for referral at least once, which serves as an indicator of the infant’s morbidity status (see Table 1). Overall, the proportions of boys and girls ill enough to be referred for care during the first 28 days of life were comparable (60.2 % and 61.1 %). A significantly higher proportion of infants were ill enough to be referred for care during the early (50.7 %) than the late neonatal period (31.3 %) [OR: 2.26, 95 % CI: 2.16 – 2.36].

The most common reasons for referral during the early neonatal period were hypothermia (22.0 %), cord infection (Level 1 (moderate to severe redness or swelling extending from the umbilicus): 14.9 %, level 2 (redness, swelling and pus coming from the umbilicus): 11.5 %), severe skin infection (7.3 %), and any two of the following; difficulty breathing, vomiting, difficulty feeding, and fever (6.2 %). During the late neonatal period the most common reasons for referral were severe skin infection (11.1 %), persistent watery stool (6.5 %), level 2 cord infection (6.2 %), and any two of the following; difficulty breathing, vomiting, difficulty feeding, and fever (6.2 %). Newborns were statistically significantly more often referred for care for fever, pneumonia, convulsions/stiffness, hypothermia, and cord infection during the early neonatal period and for having yellow body or eyes, persistent watery stool, and severe skin infection during the late neonatal period.

The proportion of boys (49.6 %) and girls (51.9 %) ill enough to require referral during the early neonatal period was similar (Table 2). There were, however, significant differences in reasons for referral. A higher proportion of boys than girls were referred for convulsions and stiffness, having a yellow body or eyes, severe skin infection, and any two of the following; difficulty breathing, vomiting, difficulty feeding, and fever (Table 2). Referral was also more often made for other (unspecified) reasons. Girls were more often referred for hypothermia and level one infection of the umbilical cord (moderate to severe redness or swelling extending from the umbilicus) (Table 2).

**Table 2:** Comparison of Referral Patterns During the Early (ENP) and Late (LNP) Neonatal Periods by Sex of the Newborn

Reason for Referral	% Boys (n)	% Girls (n)	OR (Boys/Girls)	95 % CI
	ENP (*N* = 9127)	ENP (*N* = 8544)		
	LNP (*N* = 9485)	LNP (*N* = 8852)		
% Referred				
Early Neonatal Period	49.6 % (4526)	51.9 % (4433)	0.91	0.86 – 0.96
Late Neonatal Period	32.5 % (3083)	30.0 % (2654)	1.12	1.06 – 1.20
Unconscious				
Early Neonatal Period	0.05 % (5)	0.06 % (5)	0.94	0.27 – 3.24
Late Neonatal Period	0.1 % (6)	0.03 % (3)	1.87	0.43 – 8.06
Fever				
Early Neonatal Period	1.2 % (111)	1.3 % (110)	0.94	0.74 – 1.21
Late Neonatal Period	0.8 % (74)	0.8 % (74)	0.93	0.67 – 1.30
Pneumonia				
Early Neonatal Period	0.9 % (86)	0.8 % (64)	1.26	0.91 – 1.74
Late Neonatal Period	0.6 % (55)	0.6 % (55)	0.93	0.64 – 1.36
Convulsions/ Stiffness				
Early Neonatal Period	2.4 % (218)	1.7 % (143)	1.44	1.16 – 1.78
Late Neonatal Period	1.0 % (94)	0.8 % (74)	1.19	0.89 – 1.59
Yellow Body/ Eyes				
Early Neonatal Period	2.1 % (188)	1.3 % (111)	1.60	1.26 – 2.02
Late Neonatal Period	2.7 % (257)	1.5 % (131)	1.85	1.52 – 2.26
Blood in Stool				
Early Neonatal Period	0.2 % (16)	0.3 % (22)	0.68	0.36 – 1.28
Late Neonatal Period	0.3 % (28)	0.3 % (25)	1.05	0.62 – 1.75
Persistent Watery Stool				
Early Neonatal Period	3.6 % (325)	3.6 % (308)	0.99	0.84 – 1.16
Late Neonatal Period	6.9 % (651)	6.0 % (533)	1.15	1.02 – 1.30
Hypothermia				
Early Neonatal Period	19.5 % (1782)	24.7 % (2108)	0.74	0.69 – 0.79
Late Neonatal Period	2.6 % (243)	2.8 % (243)	0.93	0.78 – 1.11
Severe Skin Infection				
Early Neonatal Period	8.0 % (732)	6.6 % (561)	1.24	1.11 – 1.39
Late Neonatal Period	11.6 % (1102)	10.5 % (926)	1.13	1.03 – 1.23
Cord Infection I				
Early Neonatal Period	14.2 % (1300)	15.7 % (1337)	0.90	0.82 – 0.97
Late Neonatal Period	1.5 % (138)	1.4 % (124)	1.04	0.82 – 1.32
Cord Infection II				
Early Neonatal Period	11.9 % (1084)	11.2 % (954)	1.07	0.97 – 1.18
Late Neonatal Period	6.0 % (564)	6.4 % (564)	0.93	0.83 – 1.04
Any Two in List				
Early Neonatal Period	6.6 % (602)	5.8 % (492)	1.16	1.02 – 1.30
Late Neonatal Period	6.4 % (602)	6.1 % (540)	1.04	0.93 – 1.17
Other				
Early Neonatal Period	3.5 % (322)	2.8 % (243)	1.25	1.04 – 1.51
Late Neonatal Period	3.2 % (307)	2.5 % (220)	1.31	1.10 – 1.56

As in the early neonatal period, there was not a meaningful statistically significant difference in the proportion of boys and girls ill enough to require referral during the late neonatal period, but there were differences between boys and girls in reason for referral (Table 2). Boys were significantly more often referred than girls for having yellow body or eyes, persistent watery stool (likely related to the common practice of early supplemental feeding, resulting in non-exclusive breastfeeding), and severe skin infection (Table 2). Additionally, boys were more often referred for care for other unspecified symptoms. Girls were not significantly more often referred than boys for any of the morbidities investigated during the late neonatal period.

Overall, care was sought for 44.1 % of infants during the neonatal period, independent of whether the infant was ill enough to be referred for care (Table 3). Care was sought significantly less often during the early (20.8 %) than the late (36.2 %) neonatal period. Although infant morbidity, defined as being ill enough to require referral, was similar when stratified by sex, care was consistently more often sought for boys regardless of time period examined. Of those taken for care, boys were, on average, taken a greater number of times (Table 3).

**Table 3:** Referral and Care-Seeking Behavior by Sex of the Newborn and Neonatal Period

Neonatal Period	Overall	Boys	Girls	OR^1^	95 % CI
Percent of Infants Seeking Care^2^ (n)	44.1 % (8242)	50.7 % (4908)	37.0 % (3334)	1.75	1.65 – 1.86
Average Number of Times Care was Sought^3^	2.84	3.01	2.60	0.41	0.31 – 0.52
% Who Sought Care, Among those Referred^4^	47.0 % (5309)	53.8 % (3128)	39.7 % (2181)	1.77	1.64 – 1.91
Early Neonatal Period					
Percent of Infants Seeking Care^5^ (n)	20.8 % (3687)	24.4 % (2239)	16.9 % (1448)	1.59	1.48 – 1.71
Average Number of Times Care was Sought^3^	1.87	1.95	1.76	0.19	0.11 – 0.27
% Who Sought Care, Among those Referred^6^	46.1 % (4126)	52.9 % (2394)	39.1 % (1732)	1.75	1.61 – 1.91
Late Neonatal Period					
Percent Infants Seeking Care^7^ (n)	36.2 % (6651)	42.5 % (4034)	29.5 % (2617)	1.76	1.66 – 1.88
Average Number of Times Care was Sought^3^	2.48	2.58	2.34	0.24	0.16 - 0.32
% Who Sought Care, Among those Referred^8^	50.8 % (2913)	56.8 % (1750)	43.8 % (1163)	1.68	1.52 – 1.86

^1^ Boys were compared to girls with girls as the reference

^2^ Overall: *N* = 18 692, Boys: *N* = 9683, Girls: *N* = 9009

^3^This variable was conditioned on care being sought

^4^ Overall: *N* = 11 304, Boys: *N* = 5815, Girls: *N* = 5489

^5^ Overall: *N* = 17 729 Boys: *N* = 9161, Girls: *N* = 8568

^6^ Overall: *N* = 8956, Boys: *N* = 4524, Girls: *N* = 4432

^7^ Overall: *N* = 18 370, Boys: *N* = 9502, Girls: *N* = 8868

^8^ Overall: *N* = 5737, Boys: *N* = 3083, Girls: *N* = 2654

Medical care was significantly more often sought than non-medical care (38.0 % and 11.5 % respectively; Δ = 26.5 %, *p*-value = <0.0001) and was significantly more often sought for boys than girls throughout the neonatal period (early neonatal period: Odds Ratio (OR) = 1.82 [95 % CI: 1.68 – 1.98], late neonatal period: OR = 1.83 [95 % CI: 1.72 – 1.95]). There were no meaningful statistically significant differences between boys and girls taken for para- or non-medical care in either the early or late neonatal periods. While the observed difference during the late neonatal period was statistically significant, there was only a 0.9 % difference between boys and girls (boys: 8.3 %, girls: 7.4 %). This difference was considered trivial, and the statistical significance driven mainly by the large sample size.

When care-seeking behavior was explored by specific cause of referral (as defined in Table 1), data were not stratified by neonatal period due to reduced sample size. The most common reasons for referral during the first 28 days of life included hypothermia (22.2 %), severe skin infection (15.2 %) and cord infection (Level 1: 15.1 %, Level 2: 15.5 %). Only about half of the newborns referred for these reasons were taken for care. Although girls were referred more often than boys for hypothermia and proportions were similar for the other reasons listed, care was more often sought for boys than girls for all referral reasons including hypothermia. Differences were significant for fever, pneumonia, watery stool, hypothermia, severe skin infection, level 1 and 2 cord infections, and any two of the following: vomiting, difficulty breathing, difficulty feeding, and fever (Table 4).

**Table 4:** Care-Seeking Behavior by Sex of the Newborn According to Cause-Specific Referral

Reason for Referral	% Referred (n)	% Who Sought Care, Given Referral	OR^1^	95 % CI
	(*N* = 18,638)			
Referral – Any Reason	60.7 % (11,304)	47.0 %	1.77	1.64 – 1.91
Boys	60.3 % (5815)	53.8 %		
Girls	61.1 % (5489)	39.7 %		
Unconscious	0.1 % (19)	79.0 %	1.50	0.15 – 14.99
Boys	0.1 % (11)	81.8 %		
Girls	0.1 % (8)	75.0 %		
Fever	1.9 % (356)	68.8 %	2.08	1.30 – 3.34
Boys	1.9 % (179)	76.5 %		
Girls	2.0 % (177)	61.0 %		
Pneumonia	1.4 % (255)	72.9 %	1.74	1.01 – 2.97
Boys	1.5 % (140)	77.9 %		
Girls	1.3 % (115)	67.0 %		
Convulsions/Stiffness	2.8 % (512)	65.8 %	1.36	0.94 – 1.95
Boys	3.1 % (300)	68.7 %		
Girls	2.4 % (212)	61.8 %		
Yellow Body/Eyes	3.0 % (556)	78.1 %	1.43	0.97 – 2.10
Boys	3.8 % (364)	80.2 %		
Girls	2.1 % (192)	74.0 %		
Watery Stool	8.9 % (1666)	49.2 %	1.34	1.12 – 1.61
Boys	9.3 % (899)	52.6 %		
Girls	8.5 % (767)	45.2 %		
Blood in Stool	0.5 % (91)	51.7 %	1.50	0.65 – 3.41
Boys	0.5 % (44)	56.8 %		
Girls	0.5 % (47)	46.8 %		
Hypothermia	22.2 % (4143)	41.4 %	1.78	1.57 – 2.02
Boys	19.8 % (1915)	48.9 %		
Girls	24.8 % (2228)	35.0 %		
Severe Skin Infection	15.2 % (2832)	52.5 %	1.51	1.31 – 1.75
Boys	16.1 % (1557)	57.2 %		
Girls	14.2 % (1275)	46.9 %		
Cord Infection – Level 1	15.1 % (2822)	45.8 %	1.72	1.48 – 2.01
Boys	14.4 % (1393)	52.6 %		
Girls	15.9 % (1429)	39.2 %		
Cord Infection – Level 2	15.5 % (2889)	48.2 %	1.65	1.44 – 1.90
Boys	15.7 % (1512)	54.1 %		
Girls	15.3 % (1377)	41.6 %		
Any Two	11.2 % (2080)	64.6 %	2.00	1.66 – 2.41
Boys	11.6 % (1118)	71.9 %		
Girls	10.7 % (962)	56.1 %		

^1^Odds ratios compare boys to girls, with girls as the reference, among those for whom care was sought

Within the Pahadi ethnic group, boys were only statistically significantly more often taken for care than girls, when referred, in families in which all prior living children were girls (ENP OR: 1.78, 95 % CI: 1.29 - 2.45 and LNP OR: 1.51, 95 % CI: 1.03 - 2.21) (Table 5). Among Madeshi families, boys were significantly more often taken for care than girls in all three family composition categories. The differences were most pronounced among neonates born to families with only prior living girls (ENP OR: 2.86, 95 % CI: 2.28 – 3.59 and LNP OR: 2.45, 95 % CI: 1.84 – 3.26) (Table 5). Additional analyses, not presented here, showed that the number of girls in a family did not impact the relationship between sex of the newborn and care-seeking.

**Table 5:** Care-Seeking Conditioned on Referral Status by Sex of the Newborn Stratified by Family Composition, Ethnicity During and Neonatal Period^1^

% Sought Care, Given Referral	% Boys (n)	% Girls (n)	OR^2^	95 % CI
Pahadi Families				
No Prior Living Children				
Early Neonatal Period (*N* = 2004)	46.4 % (243)	42.0 % (231)	1.19	0.96 – 1.49
Late Neonatal Period (*N* = 2128)	52.2 % (201)	45.8 % (154)	1.29	0.98 – 1.70
Families with Prior Boys/Boys & Girls				
Early Neonatal Period (*N* = 1769)	38.3 % (198)	37.3 % (171)	1.04	0.80 – 1.35
Late Neonatal Period (*N* = 1816)	40.9 % (137)	39.2 % (118)	1.07	0.77 – 1.50
Families with Only Prior Girls				
Early Neonatal Period (*N* = 1166)	43.3 % (142)	30.1 % (98)	1.78	1.29 – 2.45
Late Neonatal Period (*N* = 1214)	43.7 % (94)	34.0 % (67)	1.51	1.03 – 2.21
Madeshi Families				
No Prior Living Children				
Early Neonatal Period (*N* = 3804)	64.3 % (615)	49.8 % (457)	1.82	1.50 – 2.21
Late Neonatal Period (*N* = 3926)	68.1 % (431)	55.7 % (297)	1.70	1.32 – 2.17
Families with Prior Boys/Boys & Girls				
Early Neonatal Period (*N* = 5863)	52.8 % (773)	36.7 % (504)	1.92	1.63 – 2.27
Late Neonatal Period (*N* = 6067)	57.1 % (581)	41.0 % (349)	1.92	1.59 – 2.32
Families with Only Prior Girls				
Early Neonatal Period (*N* = 2736)	58.6 % (380)	33.1 (238)	2.86	2.28 – 3.59
Late Neonatal Period (*N* = 2852)	62.0 % (271)	40.0 % (154)	2.45	1.84 – 3.26

^1^The 492 newborns who died in the early neonatal period were excluded from late neonatal period analyses

^2^Odds ratios compare boys to girls, with girls as the reference

## Discussion

Among infants ill enough to require referral for care at least once during the neonatal period, less than half were taken for care. A critically important observation was that despite higher referral and mortality rates during the early neonatal period, care was more often sought during the late neonatal period. This inadequate care-seeking for neonatal illnesses is consistent with findings presented in a systematic review on neonatal care-seeking by Herbert *et al.* [31]. In South Asia, there is typically an isolation period for the mother and newborn immediately after birth, which may contribute to inhibiting care-seeking for sick newborns overall, and more markedly during the first seven days of life [32].

While there were no meaningful statistically significant sex differences in the percent of infants who were ill enough to require referral for care during the neonatal period overall or in the early/late neonatal periods, there were several significant differences in types of referral. The majority of the statistically significant differences showed increased referral among boys and were in line with biologic expectations. For example several of the categories were related to infections or infectious disease. Biologically, boys are more susceptible to infection and suffer more severe cases [4, 6, 9]. Boys were also more likely to be referred for jaundice. This is also consistent with much of the literature and aligns with increased risk factors among boys for neonatal jaundice [33, 34]. Girls were consistently more often referred for hypothermia. Hypothermia has been shown to be associated with prematurity, low birth weight, birth asphyxia, and infection [35, 36]. Although there were no differences between boys and girls in the percent born prematurely, there were significant differences in the percent born low birth weight (boys: 27.3 %, girls: 34.8 %), which may have impacted the higher proportion of girls needing referral for hypothermia. There were also consistent differences observed in warming practices favoring boys (e.g. wearing a hat, warming near a fire after bathing, etc.), however, these differences were very small and were not believed to have influenced the newborn’s well-being [19]. There may have been additional unmeasured factors influencing sex differences in hypothermia as well.

There were significant differences in care-seeking behaviors favoring boys, providing strong evidence of gender preference. Not only were boys more likely to be taken for care, but they were more likely to be taken to medical providers. Analyses investigating the relationship between care-seeking practices and cause-specific symptoms indicated that care was sought more frequently for boys than girls for every referral reason, regardless of whether boys or girls were referred more often. This study also indicated that care was more often sought among those referred for the most severe symptoms regardless of sex. It is possible that more minor symptoms spontaneously resolved and thus parents chose not to seek care. Alternatively, this may indicate that families were less likely to adhere to referral advice for more minor symptoms and were waiting, whether the newborn was a boy or girl, to seek care until symptoms became severe, or that infants (boys and girls) became severely ill rapidly, not allowing for care-seeking before death.

While these associations support the hypothesis that the excess late neonatal mortality among girls is attributable to differential care-seeking favoring boys, prior analyses adjusting for care-seeking in a multivariate model did not statistically explain the relationship between sex of the newborn and late neonatal mortality [19]. A subgroup mortality analysis among families with only prior girls, adjusting for care-seeking and ethnicity would have allowed us to determine whether care-seeking behavior was able to explain the relationship between sex of the newborn and excess late neonatal mortality among girls, as the mortality analyses indicated that this group of high risk girls was driving the observed mortality differential [19]. However, due to limitations in sample size that analysis could not be conducted with adequate precision.

Because the multivariate subgroup analysis for mortality was not possible, sex differences in care-seeking behaviors were examined according to prior sibling composition and ethnicity and were then compared to the sex-specific patterns in mortality. These analyses provided evidence of a connection between excess mortality among girls during the late neonatal period and care-seeking behavior favoring boys. In the Pahadi ethnic group, the only significant differences observed in care-seeking behaviors were between boys and girls born to families with only prior living girls. Boys in this subgroup were more often taken for care than girls throughout the neonatal period. In the Madeshi ethnic group, while care was sought more frequently for boys than girls in all family types, the difference between boys and girls in families with only prior living girls was the most pronounced. These care-seeking behaviors were consistent with observed excess late neonatal mortality among girls who were born to families with only prior living girls [19].

### Strengths and limitations

This was a large population-based study in which morbidity, referral and care-seeking data were collected prospectively throughout the neonatal period, minimizing recall bias associated with parental interviews. Data were also collected on specific reasons for referral, allowing for examination of the severity of symptoms for which care was sought, and type of care sought, providing information on when medical care was sought.

Additional information that was not collected in these trials may have enabled us to refine our conclusions. For example, information on parental reasons for seeking care would have allowed for more in-depth investigation of timing of care-seeking in relation to cause-specific referral, or barriers to care seeking and whether these differed for boys and girls.

## Conclusions

While there has been improvement in the global under-five mortality rate over the past 20 years, it is insufficient to reach the fourth Millennium Development Goal of reducing under-five mortality by two-thirds of the 1990 rate. Improvements in neonatal mortality have not been as great as those beyond the neonatal period and newborn deaths now constitute 44 % of all under-five deaths, with nearly one third of these deaths occurring in South Asia [37].

This study revealed that care-seeking, especially in the early neonatal period when mortality is highest, is inadequate regardless of newborn sex. Less than half of newborns who were ill enough to require referral were taken for care. This is likely due to cultural norms (e.g. isolation of the mother and baby immediately after birth) and environmental factors that impact delayed care-seeking (e.g. infrastructure, cost, time, distance, etc.), and must be addressed if we are to reduce neonatal mortality in South Asian populations. The timing of the decision to seek care is critical to improving neonatal survival [31].

This analysis also suggests a connection between care-seeking behaviors and excess late neonatal mortality among girls. The results indicate that different care-seeking behaviors, especially between boys and girls observed in families with only prior living girls, may play a critical role in observed excess mortality among girls. Although evidence of gender bias exists in communities in South Asia, few studies or programs aimed at reducing neonatal mortality in this region specifically address gender preference for boys and its impact on mortality or care-seeking [38–42]. Data such as ours suggest that gender inequalities in South Asia begin from the moment a child is born, and thus behavioral interventions to reduce differential care-seeking by gender must include the neonatal period in order to reduce excess mortality among girls in Nepal and throughout South Asia.
